# Impact of differing methodologies for serum miRNA-371a-3p assessment in stage I testicular germ cell cancer recurrence

**DOI:** 10.3389/fonc.2022.1056823

**Published:** 2022-12-08

**Authors:** Ailsa J. Christiansen, João Lobo, Christian D. Fankhauser, Christian Rothermundt, Richard Cathomas, Aashil A. Batavia, Josias B. Grogg, Arnoud J. Templeton, Anita Hirschi-Blickenstorfer, Anja Lorch, Silke Gillessen, Holger Moch, Jörg Beyer, Thomas Hermanns

**Affiliations:** ^1^ Department of Urology, University Hospital Zurich, University of Zurich, Zurich, Switzerland; ^2^ Department of Pathology and Molecular Pathology, University Hospital Zurich, University of Zurich, Zurich, Switzerland; ^3^ Cancer Biology and Epigenetics Group, Research Center of Portuguese Institute of Oncology (IPO) Porto, RISE@CI-IPOP Health Research Network, Portuguese Oncology Institute of Porto, Porto Comprehensive Cancer Center, Porto, Portugal; ^4^ Department of Pathology, Portuguese Oncology Institute of Porto, Porto, Portugal; ^5^ Department of Pathology and Molecular Immunology, ICBAS–School of Medicine and Biomedical Sciences, University of Porto, Porto, Portugal; ^6^ University of Zurich, Zurich, Switzerland; ^7^ Clinic for Urology, Luzerner Kantonsspital, Lucerne, Switzerland; ^8^ Department of Oncology, Kantonsspital, St. Gallen, Switzerland; ^9^ Division of Oncology/Hematology, Kantonsspital Graubünden, Chur, Switzerland; ^10^ St. Clara Research, St. Claraspital Basel and Faculty of Medicine, University of Basel, Basel, Switzerland; ^11^ Onkozentrum Hirslanden, Klinik Hirslanden, Zürich, Switzerland; ^12^ Department of Oncology, University Hospital Zurich, University of Zurich, Zurich, Switzerland; ^13^ Department of Medical Oncology, Ente Ospedaliero Cantonale (EOC) Oncology Institute of Southern Switzerland, Bellinzona, Switzerland; ^14^ Faculty of Biomedical Sciences Universita della Svizzera Italiana, Lugano, Switzerland; ^15^ Department of Medical Oncology, Inselspital, University Hospital of Bern, Bern, Switzerland

**Keywords:** miRNA - microRNA, germ cell testicular cancer, serum biomarker, method optimization, clinical implementation, disease recurrence

## Abstract

**Introduction:**

Current evidence shows that serum miR-371a-3p can identify disease recurrence in testicular germ cell tumour (TGCT) patients and correlates with tumour load. Despite convincing evidence showing the advantages of including miR-371a-3p testing to complement and overcome the classical serum tumour markers limitations, the successful introduction of a serum miRNA based test into clinical practice has been impeded by a lack of consensus regarding optimal methodologies and lack of a universal protocol and thresholds. Herein, we investigate two quantitative real-time PCR (qRT-PCR) based pipelines in detecting disease recurrence in stage I TGCT patients under active surveillance, and compare the sensitivity and specificity for each method.

**Methods:**

Sequential serum samples collected from 33 stage I TGCT patients undergoing active surveillance were analysed for miR-371a-3p *via* qRT-PCR with and without an amplification step included.

**Results:**

Using a pre-amplified protocol, all known recurrences were detected *via* elevated miR-371a-3p expression, while without pre-amplification, we failed to detect recurrence in 3/10 known recurrence patients. For pre-amplified analysis, sensitivity and specificity was 90% and 94.4% respectively. Without amplification, sensitivity dropped to 60%, but exhibited 100% specificity.

**Discussion:**

We conclude that incorporating pre-amplification increases sensitivity of miR-371a-3p detection, but produces more false positive results. The ideal protocol for quantification of miR-371a-3p still needs to be determined. TGCT patients undergoing active surveillance may benefit from serum miR-371a-3p quantification with earlier detection of recurrences compared to current standard methods. However, larger cross-institutional studies where samples are processed and data is analysed in a standardised manner are required prior to its routine clinical implementation.

## Introduction

Testicular germ cell tumours (TGCTs) are the most common solid malignancies in young-adult males worldwide (1). Definitive confirmation that a testicular mass corresponds to a germ cell tumour is only obtained after orchiectomy, following histopathological assessment. The current serum markers available in the clinic, alpha fetoprotein (AFP), human chorionic gonadotropin (HCG) and lactate dehydrogenase (LDH) show several limitations (2). They are elevated in only 60% of patients and are largely dependent on the histological composition of the tumour. Moreover, they may be elevated in other medical conditions (3). This lack of sensitivity and specificity is even more problematic for guiding treatment decisions during follow-up, since the ability of these markers to identify disease relapse is limited. Regular computed tomography (CT) scans are needed, implying exposure to radiation for a relatively young patient population, and elevated health-related costs (4). Moreover, imaging lacks the sensitivity required for early detection of occult disease.

Micro-RNA-371a-3p, (miR-371a-3p) has the potential to overcome all these issues as a robust non-invasive biomarker for implementation in the clinic. Accumulated evidence has shown, in both retrospective and prospective studies, that miR-371a-3p can reliably identify TGCTs (with the exception of teratoma) with sensitivity and specificity >90% in several contexts, from diagnosis to relapses (sensitivity reduced in this context to 82.6%) and during follow-up (5–12). The potential for clinical impact in patient management calls for its introduction in routine practice (13); however, before this is accomplished, establishment of the most robust pipeline for its determination is needed. Most efforts have used real-time quantitative PCR (qRT-PCR) for sample analysis. It is these qRT-PCR based studies that now guide the optimisation of pre-analytical variables, methods of quantification and result reporting, with the aim to provide a universal, reliable and reproducible clinical test. Optimising quantification methodologies becomes critical when defining clinically relevant cut-offs for positive values. It is yet to be determined if the most robust method relies on comparison to a healthy non-tumour population (e.g. expressed as fold change) or simply *via* a pre-defined threshold value using relative expression, for example to endogenous housekeeping MiRNAs values alone.

Most studies have focused on miR-371a-3p determination using RT-qPCR that includes pre-amplification steps in the protocol, with the aim of detecting low burden microscopic disease with the necessary sensitivity (12). In our previous study (14), focused on stage I samples, we have omitted this pre-amplification step, achieving high sensitivity and specificity for the detection of relapse. In this current work we aim to compare our previously obtained data without pre-amplification with the same RT-qPCR protocol plus an additional pre-amplification step, performed on the same clinical samples.

## Materials and methods

### Blood collection and serum processing

Whole blood was collected and processed as previously described (14). In brief, we prospectively collected 143 serum samples from 33 men with stage I testicular GCT undergoing active surveillance after orchiectomy without adjuvant chemotherapy who provided written informed consent and were registered in the Swiss Austrian German Testicular Cancer Cohort Study (SAG TCCS; NCT02229916) (15). Samples selected for miRNA analysis were prioritised based on earliest consecutive comprehensive set received into the registry. Clinical features of patients with proven recurrence including histopathology, details of standard methods of recurrence and site of recurrence are detailed in **Supplementary Table 1**. The cohort with known recurrence consisted of 8 seminoma and 2 non-seminoma patients, as determined *via* histopathological analysis. Samples from 10 men, who underwent orchiectomy but had non-GCT histology, were used as controls.

Serum samples were collected at each patient visit during standard follow-up as described in (14). Whole blood was processed as described and the resulting serum was aliquoted in an RNase free workspace into cryovials. Aliquots were subsequently stored at -80°C.

### RNA extraction

For all analyses, RNA was extracted from 200 μL of serum using the miRNeasy serum kit (Qiagen) into a final volume of 100 μL nuclease free water as described previously (12). All isolated RNA was stored at -80°C. RNA yield was maximised with the addition of MS2 carrier RNA (Roche, final concentration 1.25 μg ml^−1^) to Qiazol prior to isolation and the exogenous spike-in miRNA cel-miR-39-3p (5.6 × 10^8^ copies) was used as an initial quality control for extraction efficiency. All samples underwent quality control analysis prior to subsequent target miRNA qRT-PCR analyses (12, 16). Quality control analysis included quantification of cel-miR-39-3p, the endogenous housekeeper miR-30b-5p and the haemolysis control miRNAs miR-451a and miR-23a-3p as previously described (12). Consistency of extraction was acceptable for all samples analysed (14). Haemolysis assessment was performed by calculating the delta Ct values for miR-23a-3p minus miR-451a, detailed in (14).

### Real-time PCR analysis with and without prior amplification

Five μL of RNA was reverse transcribed using the TaqMan miRNA reverse transcription kit (Life Technologies, Paisley, UK) using the miRNA-specific stem-loop primer from the relevant TaqMan miRNA assay kit (Life Technologies), as per the manufacturer’s instructions. The final volume of 15 μL for each reaction underwent reverse transcription using a Eppendorf Mastercycler pro S thermocycler at 16 °C for 30 min, 42 °C for 30 min, followed by a final step of 85 °C for 5 min, as described previously (14, 17). qRT-PCR was initially performed on un-amplified singleplex cDNA using TaqMan Fast advanced Master Mix as per the manufacturer’s instructions, using primer probe sets described in **Supplementary Table 2**, and run on an Applied Biosystems ViiA 7 System (14). RT-PCR conditions were to hold at 95 °C for 2 mins, followed by 45 cycles of 95 °C for 1 second and 60 °C for 20 seconds. To exclude non-specific amplification, a non-template control was run for each assay. In all cases, no product was detectable. A standard positive sample (miRNA extracted from the TCam-2 seminoma cell line, a kind gift from Professor Matthew Murray, University of Cambridge, United Kingdom) was also routinely assayed as an internal experimental control. The miR-371a-3p results were not communicated to the treating clinicians and did not influence follow-up management. Mir-30b-5p and miR-371a-3p cDNA was also pre-amplified (12 cycles) using 2× TaqMan PreAmp Master Mix (Thermo Fisher Scientific) as per the manufacturers’ instructions. Quantitative reverse transcription-polymerase chain reaction (RT-qPCR) was also then run using this pre-amplified cDNA. In each plate, appropriate positive [seminoma-like cell line TCam-2 (18)] and negative (no template control) controls were included.

### Statistical analysis

In our previous study, relative expression of the unamplified target miRNA (miR-371a-3p) was calculated *via* standard delta Ct calculations compared to the housekeeper miR-30b-5p (rE = 2^-(ΔCt) where ΔCt = Ct (miR-371a-3p) – Ct (miR-30b-5p) as previously described (19). In this study, both un-amplified and pre-amplified samples were analysed and the fold change of miR-371a-3p was calculated relative to the mean relative expression of our control (non-GCT group). Calculating fold change to our control non-GCT cohort was now possible as we assumed samples with undetected miR-371a-3p to take the maximum cycle number (ie. Ct=45). Relative expression was calculated as RE =2^-(ct (miR-371a-3p) -ct (MiR-30b-5p)) with fold change calculated as FC = RE (sample)/Mean (RE control group). ROC curve, sensitivity and specificity (with 95% confidence intervals) were computed for assessing diagnostic performance of un-amplified and pre-amplified protocols. ROC curves were generated for both Pre-amplification and No-Amplification analyses using all known negative values taken from non-recurrence samples and the known positive miRNA expression FC values for recurrence patients at the point at which recurrence occurred, taking into account also all false negative and false positive values acquired.

## Results

**Figure 1:**
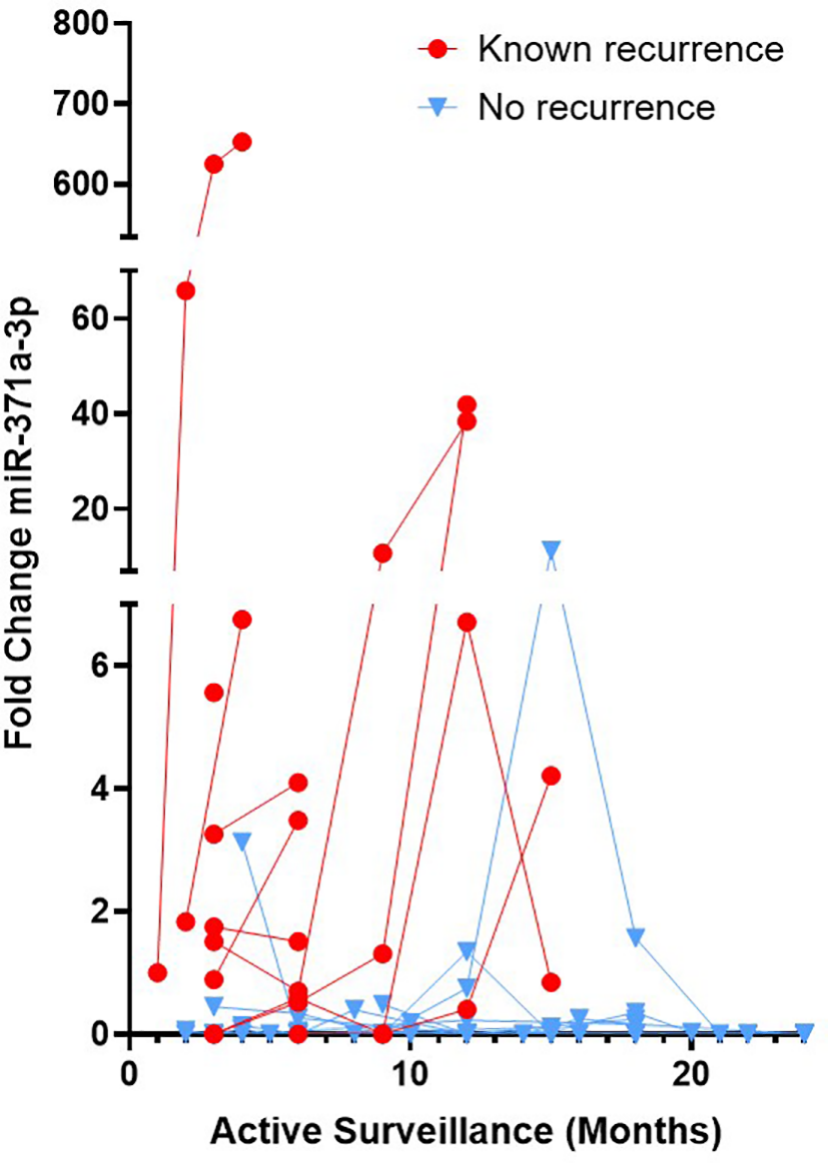
TGCT Patients with recurrence show elevated serum miR-371a-3p when adopting a protocol with pre-amplification. Sequential serum samples from Stage 1 TGCT patients undergoing active surveillance were analysed for serum miR-371a-3p *via* RT-PCR including a cDNA pre-amplification step. Patients with known recurrence (red dots) and known non-recurrence (blue triangles) are shown. FC calculated *via* relative expression to the endogenous housekeeper miR-30b-5p and to a control, no tumour cohort. FC>1.1 considered a positive value.

When analysing patient samples with a pre-amplification step included in the protocol, in our cohort of 33 patients, all 10 patients with known clinical recurrences exhibited elevated levels of miR-371a-3p when compared to our control cohort without GCT (FC>1.1) **Figure 1**. Three patients without known recurrences also displayed elevated miR-371a-3p FC levels, 2 of which subsequently dropped to FC<1.1 during further follow-up analyses. Of these three patients, none of them developed clinical evidence of a relapse. This suggests we detected 3 false positives among 23 known non-recurrence patients when adopting a protocol that includes a pre-amplification step **Table 1**. Recurrences detected *via* serum miR-371a-3p using a pre-amplification protocol were found at a median of 92 days (IQR = 82-300 days, Range = 41-458 days) as compared to standard biomarker analysis (AFP, LDH, HCG) and imaging at a median of 200 days (IQR = 140-395 days, Range = 89-458 days). This corresponds to an earlier detection range using miRNA analysis with pre-amplification of up to 9 months (**Table 1**).

**Table 1:** Comparison of methodologies and data analysis pipelines for detection of disease recurrence in patients with Stage I TGCT.

Method for recurrence detection	Data analysis pipeline	Recurrence detected (of 10 known recurrence cases)	False positive rate (of 23 non-recurrence cases)	Range detection time benefit cf Standard
RT-PCR Non-amplified*	Relative expression	100 %	4.4 %	0-5 months
RT-PCR Non-Amplified	Fold change	70 %	0 %	0-3 months
RT-PCR Pre-Amplified	Fold change	100 %	13 %	0-9 months
Standard Clinical Markers	AFP/HCG/LDH MRI/CT	100 %	0 %	N/A

* Refers to data presented in (14).

**Figure 2:**
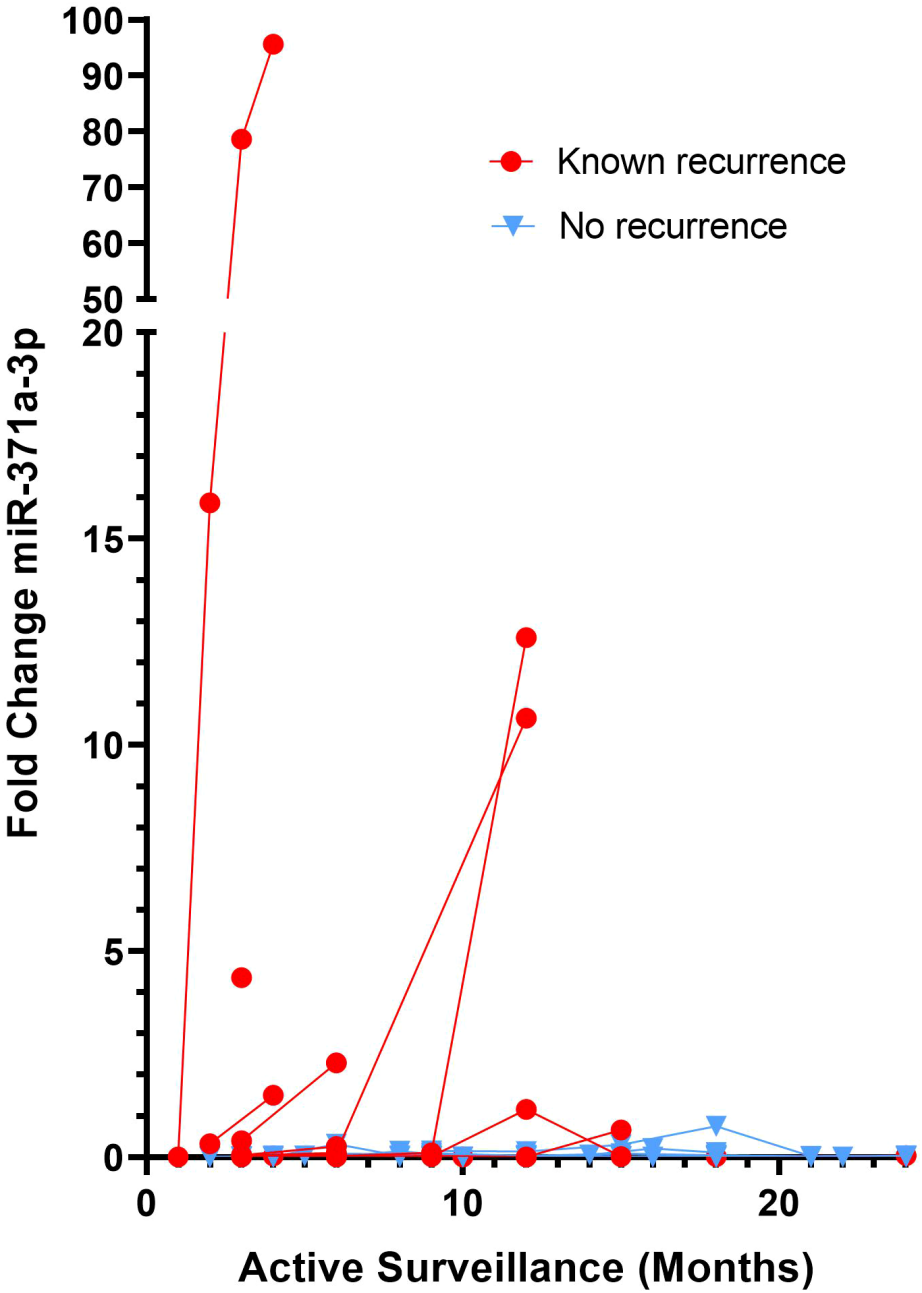
Seven of ten TGCT Patients with recurrence show elevated serum miR-371a-3p when adopting a protocol without pre-amplification. Sequential serum samples from Stage 1 TGCT patients undergoing active surveillance were analysed for serum miR-371a-3p *via* RT-PCR without a cDNA pre-amplification step. Patients with known recurrence (red dots) and known non-recurrence (blue triangles) are shown. FC calculated *via* relative expression to the endogenous housekeeper miR-30b-5p and to a control, no tumour cohort. FC>1.1 considered a positive value.

**Figure 3:**
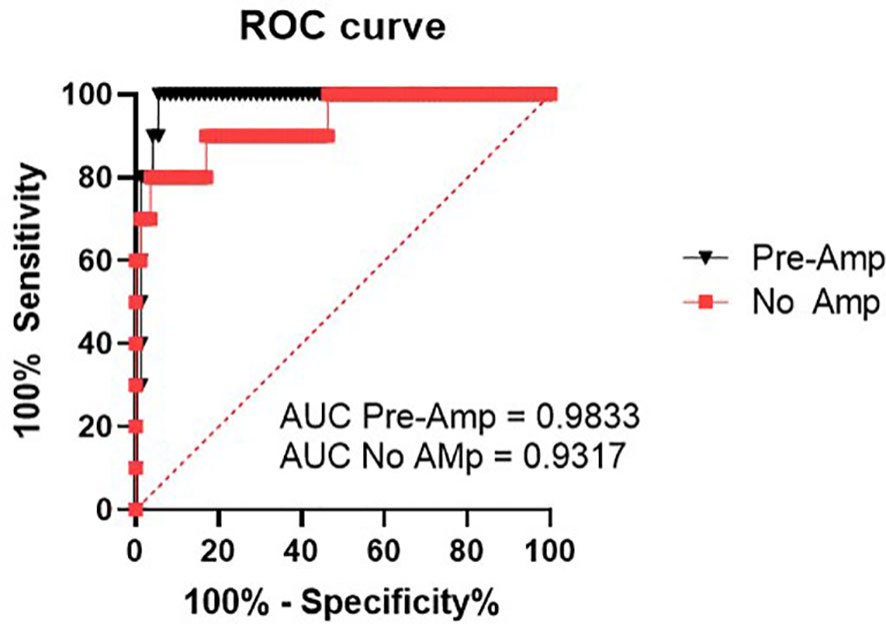
ROC curves generated using all no-recurrence values as negative samples and the endpoint known recurrence values as defined by detection *via* standard methods (imaging/HCG,LDH,AFP), including all false negative and false positive values.

**Figure 4:**
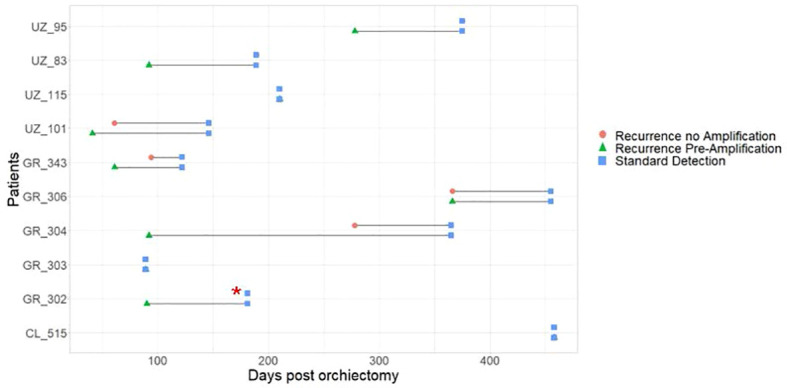
Comparison of recurrence detection during active surveillance of Stage I TGCT patients. Data shown compares the time of recurrence as detected *via* elevated serum MiR-371a-3p (Fold Change) *via* Non-Amplified (Red circle), Pre-Amplified (Green triangle) and *via* current parameters established in clinical practice (Blue square) post orchiectomy. Red asterix (*) indicates recurrence was not detected in known recurrence patient when using non-amplified protocol.

In our previous study (14), all samples had been quantified without pre-amplification and data was expressed as relative expression to the housekeeper MiR-30b-5p. In the present study, we re-analysed this data and assumed non-detected values to take a maximum cycle number (Ct) of 45. Therefore, in this study, we are now able to evaluate expression of miR-371a-3p as a fold change to our control group without GCT. This enables us to do a direct comparison between the two protocols, one with, and one without pre-amplification. When expressed as FC to our control without GCT group, 7 of 10 known recurrence patients exhibited elevated FC miR-371a-3p levels (FC>1.1) when compared to our control without GCT (**Figure 2**). One patient that had miR-371a-3p detected above the FC threshold subsequently dropped to below the FC cut off during follow-up. Three patients with known recurrence were not detected. Of the 7 patients where recurrence was detected, recurrence detection occurred at a median of 189 days (IQR = 89-366 days, Range = 61-375 days). This corresponds to a benefit range of detection between serum miRNA analysis without amplification when compared to standard methods of 0-3 months (**Table 1**). No patients without known recurrence showed elevated FC miR-371a-3p levels, indicative that no false positives were detected (**Table 1**). ROC curves for both pre-amplification and no-Amplification analyses were generated using all known negative values taken from non-recurrence samples and the known positive miRNA expression FC values for recurrence patients at the point at which recurrence was detected by standard methods (**Figure 3**). Pre-amplified analysis gave an AUC of 0.983 while non-amplified AUC was 0.932. With an FC cut-off of >1.1 for pre-amplified analysis, sensitivity and specificity was found to be 90.0% and 94.4% respectively, whereas without amplification sensitivity dropped to 60% however specificity was found to be 100% (**Table 2**). Differences in recurrence detection when comparing qRT-PCR miR-371a-3p quantification and standard clinical parameters are shown in **Figure 4**. Pre-Amplification detected recurrence 108 days earlier than standard methods, while a no amplification protocol detected only 11 days earlier and failed to identify 3 known recurrence patients. When comparing miR-371a-3p quantification *via* relative expression to the endogenous housekeeper miR-30b-5p with standard detection methods (**Supplementary Figure 1**), we found non-amplification quantification *via* analysis of relative expression gave a median detection benefit range of 0-151 days (14). A summary of the differing methodologies is shown in **Table 1** highlighting % known recurrences detected, number of false positives per methodology and range of benefit time until recurrence was detected compared to standard methods.

**Table 2:** Sensitivity and specificity calculated from the above ROC curve using the endpoint known recurrence values as defined by detection *via* standard methods (imaging/HCG,LDH,AFP) and a FC >1.1 cutoff.

Method	Sensitivity	Specificity
Pre-Amplification	90% CI 95% (59.6-99.5%)	94.4% CI 95% (86-6%-97.2%)
No Amplification	60% CI 95% (31.27-83.18%)	100% CI 95% (95.5-100%)

## Discussion

Although showing great promise in pre-clinical studies, serum miRNA-371a-3p analysis has not yet been incorporated into standard clinical practice for either initial diagnosis or disease progression monitoring in TGCT. This is largely due to discrepancies between investigating groups who have not yet reached a consensus regarding optimal protocols for pre-analytical sample handling, miRNA extraction methodologies, protocols for RT-PCR quantification, downstream data analysis workflows, or a clear definition of appropriate thresholds for positivity. In this present work we start to address these shortcomings and compared protocols with and without pre-amplification of cDNA prior to quantification *via* RT-PCR, and investigated two downstream data analysis workflows where fold change to a control group of non-tumour patients was used and compared to a method investigating relative expression above a defined threshold.

Although our study is limited by the relatively small patient group analysed, it can be taken in the context of prior larger cohort studies that have utilised protocols that incorporated pre-amplification prior to qRT-PCR analysis. Indeed, a recent prospective, multicentric study that investigated the use of serum mir-371a-3p analysis for diagnosis of TCGT was undertaken in a much larger cohort (616 patients) and showed convincingly that miR-371a-3p out-performed currently utilised serum biomarkers (9). This was evident for initial TCGT diagnosis but also demonstrated serum miR levels were significantly associated with a variety of relevant clinical parameters including clinical stage, primary tumour size, response to treatment and remission (9). The methodology described by Dieckmann et al. (MIR371 test) included pre-amplification similar to our protocol described herein and in a TCGT diagnosis setting exhibited the same sensitivity and specificity (sensitivity of 90.1%, a specificity of 94.0%) as we found in a recurrence setting when a pre-amplification step was incorporated. Although tempting to speculate the significance of similar specificities and sensitivities between the two studies and differing settings, it is important to note that our study is limited by the relatively small patient group analysed and should be considered with caution. Nonetheless, the promising sensitivity and specificity values obtained in the current study when incorporating a pre-amplification step, not only suggest serum miRNA-371a-3p analysis with pre-amplification could prove valuable in a clinical active surveillance phase for disease recurrence detection, it also suggests our small cohort reflects the situation of larger cohort studies, validating its use for our comparative protocol study.

Indeed, in our study, when incorporating a pre-amplification step, disease relapse could be identified in all 10 known recurrence patients (10/10), for up to 9 months earlier than when using standard methods currently adopted in clinical practice. When samples were analysed without pre-amplification, only 7 of the 10 known recurrence patients were identified *via* elevated serum miR-371a-3p levels, with a benefit of up to 3 months when compared to standard methods. Earlier detection of relapse would allow for timely treatment, namely for giving chemotherapy earlier and potentially with fewer cycles. This is relevant given the large epidemiological studies showing the short- and long-term side effects of platinum-based chemotherapy in such young TGCT survivors (including second malignancies, cardiovascular disease, etc), which are dependent on dose and number of cycles (20).

Increased sensitivity when a pre-amplification step is included provides the benefit of earlier, more reliable detection of recurrence; however it is important to note that there are also issues related to pre-amplification protocols. Compared to classical serum tumour markers, which were elevated at relapse in stage I patients on surveillance in only 38% of patients in this series, pre-amplified miR-371a-3p was elevated in 94% (21). However, pre-amplification requires an extra step in the protocol, which increases hands-on time for the operator and increases the cost of the test. Pre-amplification reactions are more prone to non-specific amplifications in RT-qPCR by increasing cycling time and detecting negligible levels of the desired target in the background, including those related to small contaminations. Our data corroborates this by showing an increase in false positive results (3/23 compared to 0/23 without pre-amplification). Since TGCTs are a model of curable disease, it may be argued that false positives should be avoided in this tumour model, to avoid giving unnecessary toxic treatment, at the expense of detecting relapse later in time during follow-up. However, when determining the optimal protocol for introduction of serum miR371a-3p analysis into clinical practice, it is also important to consider how treating clinicians may act following receipt of a positive serum miRNA-371a-3p value including decision to treat and the chemotherapy protocol administered. It is most likely, upon detection of a positive serum miRNA test, that rather than immediate treatment, additional imaging (for example, CT or PET) would be performed to corroborate the serum miRNA findings. Adopting this as clinical practice would mitigate the potential for false positive overtreatment that would likely occur when using a protocol that incorporates a pre-amplification step. When considering this aspect of assay development, it is conceivable that prioritising sensitivity (and therefore potentially earlier detection) would prove beneficial to a more cautious non-amplified protocol with lower sensitivity but higher specificity. As we approach adoption of miRNAs into clinical implementation it is important to discuss and highlight differences in protocols and how these affect the way results are reported to clinicians. Such discussions should assist and guide how clinicians react to serum miRNA results, highlighting the relevance of our present work.

Another consideration relates to thresholds of detection of miR-371a-3p. While pre-amplification brings the commonly used Ct values to a more comfortable range of detection, the protocol without pre-amplification raises insecurity in dealing with very high Ct value ranges, where RT-qPCR reading is less precise and reliable. Differing data analysis workflows (fold change compared to relative expression to an endogenous housekeeper) also impacts upon stringency of test results. This was observed when comparing our unamplified data where relative expression quantification identified all 10 of the recurrence patients, however also identified 1 false positive patient. This contrasted a workflow using fold change analysis compared to a known tumour negative cohort (where an undetected value was assigned a Ct of 45 for undetected cases) which failed to identify 3 known recurrence patients however falsely identified 1 patient. Another issue relates to the establishment of a FC threshold of positivity. In our study a FC>1.1 was considered, however if a FC>1.5 was established, pre-amplification would still result in all known recurrences being detected (10/10) however 1 patient would be detected 3 months later. False positives would be then reduced to 2/23. For the protocol without amplification, one fewer patient would be detected to recur (ie. 6/10) and no false positive cases would occur. Setting the appropriate thresholds for positivity will be key to trading off the benefits of early detection with the disadvantages of the detection of false positives and unnecessary over treatment.

To conclude, the ideal protocol for quantification of miR-371a-3p still needs to be determined. Larger cross-institutional studies where samples are processed and data is analysed in a standardised manner are required before serum miR-371a-3p analysis for TGCT can be implemented into clinical practice (22). Additional variables not discussed herein, including sample type (plasma vs. serum, etc.), method of extraction (beads vs. columns, etc.), sample quality control measures (hemolysis and extraction efficacy) (23), and PCR methodology (regular real-time versus digital droplet PCR) (24, 25) will also most likely impact optimised protocol development and should be harmonised for creating a universal, robust, clinical test for guiding treatment decisions.

## Data availability statement

The original contributions presented in the study are included in the article/**Supplementary Material**. Further inquiries can be directed to the corresponding author.

## Ethics statement

The studies involving human participants were reviewed and approved by the local ethical committee of St. Gallen (EKSG 13/08/L). The patients/participants provided their written informed consent to participate in this study.

## Author contributions

Author contributions are as follows. Conception and design: AC, JL, CF, JB, TH, CR. Acquisition of Data: AC, JG, CF, TH, CR, RC, SG, AL, AT and AH-B. Analysis and Interpretation of Data: AC, JL. Drafting of Manuscript: AC, JL. Manuscript revision: All Authors. Statistical Analysis: AC, AB, JL. Obtaining funding: JB, TH, CF. Administrative, technical assistance: JG, AB. Supervision: AC, JL. All authors contributed to the article and approved the submitted version.

## References

[1] Trabert B Chen J Devesa SS Bray F McGlynn KA. International patterns and trends in testicular cancer incidence, overall and by histologic subtype, 1973-2007. Andrology (2015) 3:4–12. doi: 10.1111/andr.293 25331326 PMC4410839

[2] Murray MJ Huddart RA Coleman N. The present and future of serum diagnostic tests for testicular germ cell tumours. Nat Rev Urol (2016) 13:715–25. doi: 10.1038/nrurol.2016.170 27754472

[3] Gilligan TD Seidenfeld J Basch EM Einhorn LH Fancher T Smith DC. American Society of clinical oncology clinical practice guideline on uses of serum tumor markers in adult males with germ cell tumors. J Clin Oncol (2010) 28:3388–404. doi: 10.1200/JCO.2009.26.4481 20530278

[4] Mir MC Pavan N Gonzalgo ML. Current clinical applications of testicular cancer biomarkers. Urol Clin North Am (2016) 43:119–25. doi: 10.1016/j.ucl.2015.08.011 26614034

[5] Syring I Bartels J Holdenrieder S Kristiansen G Müller SC Ellinger J. Circulating serum miRNA (miR-367-3p, miR-371a-3p, miR-372-3p and miR-373-3p) as biomarkers in patients with testicular germ cell cancer. J Urol (2015) 193:331–7. doi: 10.1016/j.juro.2014.07.010 25046619

[6] van Agthoven T Eijkenboom WMH Looijenga LHJ. microRNA-371a-3p as informative biomarker for the follow-up of testicular germ cell cancer patients. Cell Oncol (2017) 40:379–88. doi: 10.1007/s13402-017-0333-9 PMC5537315 28612337

[7] Spiekermann M Belge G Winter N Ikogho R Balks T Bullerdiek J. MicroRNA miR-371a-3p in serum of patients with germ cell tumours: evaluations for establishing a serum biomarker. Andrology (2015) 3:78–84. doi: 10.1111/j.2047-2927.2014.00269.x 25187505

[8] Dieckmann K-P Spiekermann M Balks T Ikogho R Anheuser P Wosniok W. MicroRNA miR-371a-3p - a novel serum biomarker of testicular germ cell tumors: Evidence for specificity from measurements in testicular vein blood and in neoplastic hydrocele fluid. Urol Int (2016) 97:76–83. doi: 10.1159/000444303 26989896

[9] Dieckmann K-P Radtke A Geczi L Matthies C Anheuser P Eckardt U. Serum levels of MicroRNA-371a-3p (M371 test) as a new biomarker of testicular germ cell tumors: Results of a prospective multicentric study. J Clin Oncol (2019) 37:1412–23. doi: 10.1200/JCO.18.01480 PMC6544462 30875280

[10] Dieckmann K-P Spiekermann M Balks T Flor I Löning T Bullerdiek J. MicroRNAs miR-371-3 in serum as diagnostic tools in the management of testicular germ cell tumours. Br J Cancer (2012) 107:1754–60. doi: 10.1038/bjc.2012.469 PMC3493876 23059743

[11] Dieckmann K-P Radtke A Spiekermann M Balks T Matthies C Becker P. Serum levels of MicroRNA miR-371a-3p: A sensitive and specific new biomarker for germ cell tumours. Eur Urol (2017) 71:213–20. doi: 10.1016/j.eururo.2016.07.029 27495845

[12] Murray MJ Bell E Raby KL Rijlaarsdam MA Gillis AJM Looijenga LHJ. A pipeline to quantify serum and cerebrospinal fluid microRNAs for diagnosis and detection of relapse in paediatric malignant germ-cell tumours. Br J Cancer (2016) 114:151–62. doi: 10.1038/bjc.2015.429 PMC4815809 26671749

[13] Badia RR Abe D Wong D Singla N Savelyeva A Chertack N. Real-world application of pre-orchiectomy miR-371a-3p test in testicular germ cell tumor management. J Urol (2021) 205:137–44. doi: 10.1097/JU.0000000000001337 32856980

[14] Fankhauser C Christiansen A Rothermundt C Cathomas R Wettstein M Grossmann N. Detection of recurrences using serum miR-371a-3p during active surveillance in men with stage I testicular germ cell tumours. Br J Cancer (2022). 126(8):1140–4. doi: 10.1038/s41416-021-01643-z PMC9023438 34912073

[15] Rothermundt C Thurneysen C Cathomas R Müller B Mingrone W Hirschi-Blickenstorfer A. Baseline characteristics and patterns of care in testicular cancer patients: first data from the Swiss Austrian German testicular cancer cohort study (SAG TCCS). Swiss Med Wkly (2018) 148:w14640. doi: 10.4414/smw.2018.14640 30044478

[16] Bell E Watson HL Bailey S Murray MJ Coleman N. A robust protocol to quantify circulating cancer biomarker MicroRNAs. Methods Mol Biol (2017) 1580:265–79. doi: 10.1007/978-1-4939-6866-4_18 28439839

[17] Murray MJ Halsall DJ Hook CE Williams DM Nicholson JC Coleman N. Identification of microRNAs from the miR-371~373 and miR-302 clusters as potential serum biomarkers of malignant germ cell tumors. Am J Clin Pathol (2011) 135:119–25. doi: 10.1309/AJCPOE11KEYZCJHT 21173133

[18] de Jong J Stoop H Gillis AJM Hersmus R van Gurp RJHLM van de Geijn G-JM. Further characterization of the first seminoma cell line TCam-2. Genes Chromosomes Cancer (2008) 47:185–96. doi: 10.1002/gcc.20520 18050305

[19] Livak KJ Schmittgen TD. Analysis of relative gene expression data using real-time quantitative PCR and the 2– ΔΔCT method. Methods (2001). 25(4):402–8. doi: 10.1006/meth.2001.1262 11846609

[20] Hellesnes R Myklebust TÅ Fosså SD Bremnes RM Karlsdottir Á Kvammen Ø. Testicular cancer in the cisplatin era: Causes of death and mortality rates in a population-based cohort. J Clin Oncol (2021) 39:3561–73. doi: 10.1200/JCO.21.00637 34388002

[21] Lobo J Leão R Gillis AJM van den Berg A Anson-Cartwright L Atenafu EG. Utility of serum miR-371a-3p in predicting relapse on surveillance in patients with clinical stage I testicular germ cell cancer. Eur Urol Oncol (2021) 4(3):483–91. doi: 10.1016/j.euo.2020.11.004 33288479

[22] Murray MJ Watson HL Ward D Bailey S Ferraresso M Nicholson JC. “Future-proofing” blood processing for measurement of circulating miRNAs in samples from biobanks and prospective clinical trials. Cancer Epidemiol Biomarkers Prev (2018) 27:208–18. doi: 10.1158/1055-9965.EPI-17-0657 PMC5812437 29254935

[23] Myklebust MP Rosenlund B Gjengstø P Bercea BS Karlsdottir Á Brydøy M. Quantitative PCR measurement of miR-371a-3p and miR-372-p is influenced by hemolysis. Front Genet (2019) 10:463. doi: 10.3389/fgene.2019.00463 31191602 PMC6539204

[24] Myklebust MP Thor A Rosenlund B Gjengstø P Karlsdottir Á Brydøy M. Serum miR371 in testicular germ cell cancer before and after orchiectomy, assessed by digital-droplet PCR in a prospective study. Sci Rep (2021) 11:15582. doi: 10.1038/s41598-021-94812-2 34341387 PMC8329070

[25] Sequeira JP Lobo J Constâncio V Brito-Rocha T Carvalho-Maia C Braga I. DigiMir test: Establishing a novel pipeline for MiR-371a quantification using droplet digital PCR in liquid biopsies from testicular germ cell tumor patients. Front Oncol (2022) 12:876732. doi: 10.3389/fonc.2022.876732 35756620 PMC9226402

