# Supplementary material for: Impact of differing methodologies for serum miRNA-371a-3p assessment in stage I testicular germ cell cancer recurrence
Source: Front Oncol. 2022 Dec 8;12:1056823. doi: 10.3389/fonc.2022.1056823 (PMC9773982; doi:10.3389/fonc.2022.1056823)
Supplement: Supplementary file 1 [file DataSheet_1.docx]

**Supplementary Material**

**Supplementary Figure 1**

**
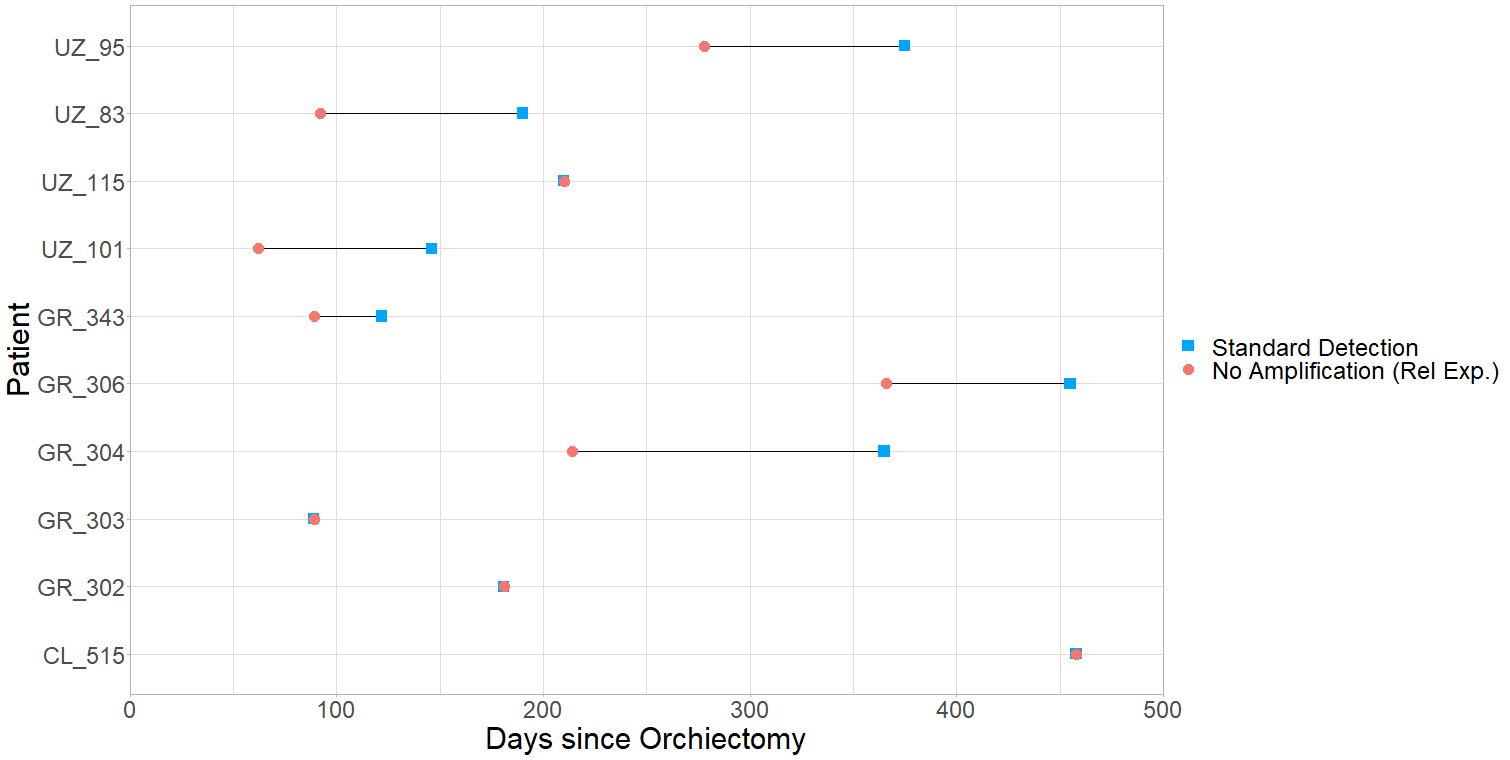
**

**Supplementary Figure 1**: Shows comparison between serum miRNA analysis as determined without pre-amplification and analysed via relative expression (Positive values Rel Exp > 0.0001). Red dots are time post orchiectomy as determined via serum miRNA Relative expression to endogenous housekeeper miR-30b-5p. Blue squares are time post orchiectomy until recurrence detection via Standard clinical methods.

**
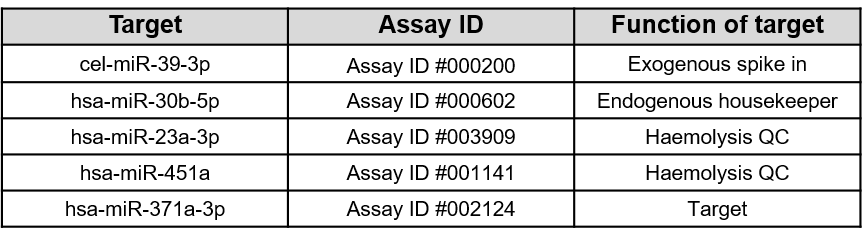

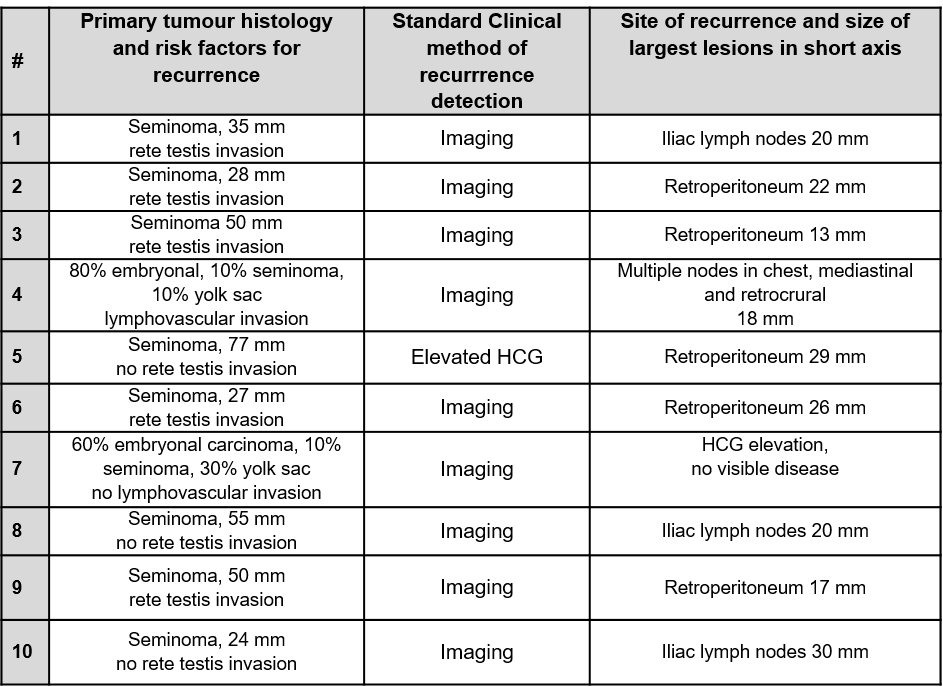
**

**Supplementary Table 2** Target, Assay ID and function of target of miRNAs investigated

**Supplementary Table 1**: Clinical cohort characteristics of patients with known recurrence. Histology of primary tumour, method of conventional recurrence detection and site and size of recurrence lesions.

Abbreviation: HCG human chorionic gonadotrophin.
